# Sex-dependence of synaptic depression induced by activation of metabotropic glutamate receptors in rat hippocampus

**DOI:** 10.1177/23982128231223579

**Published:** 2024-01-29

**Authors:** Liam T. Ralph, John Georgiou, Graham L. Collingridge, Patrick Tidball

**Affiliations:** 1 Department of Physiology, University of Toronto, Toronto, ON, Canada; 2 Lunenfeld-Tanenbaum Research Institute, Mount Sinai Hospital, Sinai Health System, Toronto, ON, Canada; 3 TANZ Centre for Research in Neurodegenerative Diseases, University of Toronto, Toronto, ON, Canada

**Keywords:** Sex difference, 3,5-dihydroxyphenylglycine, metabotropic glutamate receptors, long-term depression, synaptic plasticity, DHPG, LTD

## Abstract

The modulation of synaptic efficacy by group I metabotropic glutamate receptors is dysregulated in several neurodevelopmental and neurodegenerative disorders impacting cognitive function. The progression and severity of these and other disorders are affected by biological sex, and differences in metabotropic glutamate receptor signalling have been implicated in this effect. In this study, we have examined whether there are any sex-dependent differences in a form of long-term depression of synaptic responses that is triggered by application of the group I metabotropic glutamate receptor agonist 3,5-dihydroxyphenylglycine (DHPG). We studied DHPG-induced long-term depression at the Schaffer collateral-commissural pathway in area CA1 of hippocampal slices prepared from three separate age groups of Sprague Dawley rats. In both juvenile (2-week-old) and young adult (3-month-old) rats, there were no differences between sexes in the magnitude of long-term depression. However, in older adult (>1-year-old) rats, DHPG-induced long-term depression was greater in males. In contrast, there were no differences between sexes with respect to basal synaptic transmission or paired-pulse facilitation in any age group. The specific enhancement of metabotropic glutamate receptor–dependent long-term depression in older adult males, but not females, reinforces the importance of considering sex as a factor in the study and treatment of brain disorders.

The incidence and severity of many brain disorders are influenced by biological sex and age. For instance, Fragile X Syndrome is more common in males, whereas Alzheimer’s disease is more common in females. Understanding how biological sex impacts brain function at different ages is therefore extremely important from a therapeutic perspective. In this context, previous studies have identified sex-dependent differences in pathophysiological processes involving group I metabotropic glutamate receptors (mGluRs). For example, male mice are more sensitive to treatment with an mGlu5 receptor antagonist in models of both Alzheimer’s disease (Abd-Elrahman et al., 2020) and Huntington’s disease (Li et al., 2022), suggesting that mGluR signalling may play a greater role in the progression of these diseases in males, while other mechanisms may dominate in females.

A widely studied biological process that is triggered by the activation of group I mGluRs is long-term depression (LTD), a form of synaptic weakening. Using female rats, we showed that the brief application of the group I mGluR agonist 3,5-dihydroxyphenylglycine (DHPG) results in LTD of synaptic transmission at the Schaffer collateral-commissural pathway (SCCP) in the CA1 region of the hippocampus (Palmer et al., 1997). Since then, DHPG-LTD has been extensively studied largely in male rodents, while no direct comparison between the sexes has been reported. Therefore, in this study, we have compared DHPG-LTD, as well as basal synaptic properties, in the SCCP-CA1 of male and female rats in three age groups. We find that the magnitude of DHPG-LTD is enhanced in older male but not female rats.

Juvenile, young adult and older adult (0.5-, 3- and 12-to-16-month-old, respectively) male and female Sprague Dawley (SD 001) rats were bred in-house within the University Health Network facilities with breeding pairs replaced every 6 months. Breeding pairs were initially obtained from Charles River (Quebec, Canada). Experimental animals were kept under standard housing conditions in static plastic cages (26 cm W × 20 cm H × 48 cm D) in pairs of the same sex on a 12-/12-h light/dark cycle beginning at 07:00. All experiments were performed in accordance with the University Health Network animal use protocol and oversight from The Centre for Phenogenomics. Rats were euthanized by decapitation under isoflurane anaesthesia, and brains were rapidly extracted and placed in ice-cold artificial cerebrospinal fluid (ACSF) saturated with 95% O_2_ and 5% CO_2_. For juvenile rats, slices were prepared in standard ACSF composed of (in mM): 124 NaCl, 10 D-Glucose, 24 NaHCO_3_, 3 KCl, 1.25 NaH_2_PO_4_, 1 MgSO_4_ and 2 CaCl_2_. A modified ACSF was used for preparation of slices from both young adult and older adult rats, which contained 205 sucrose in the place of NaCl, and divalent cations were altered to 5 MgSO_4_ and 0.5 CaCl_2_. This protective sucrose-based cutting solution was used to maximise the quality of adult brain slices, which are more sensitive than juvenile slices to excitotoxic damage during the slicing procedure. Transverse slices (400 µm in thickness) of dorsal hippocampus were cut using a vibratome as previously described (Tidball et al., 2017). Slices from all age groups were then allowed to recover in standard ACSF at room temperature. For this study, the CA3 region was not removed from slices. Following recovery for > 1.5 h, slices were transferred to a submerged recording chamber where they were continuously perfused with standard ACSF at a rate of 2.5 mL/min and maintained at 30°C. Extracellular field potentials were recorded from the CA1 stratum radiatum in response to 0.033-Hz constant current stimulation of the SCCP. A maximum of two slices per animal and a minimum of five animals were used in any experimental group. The reported n values represent the number of slices and the number of animals (slices/animals). Statistics were performed using the number of slices. Sample sizes were chosen based on our previous experience of slice electrophysiology, availability of experimental animals and reference to prior DHPG-LTD experiments (e.g. Tidball et al., 2017). Within each age group, recordings from male and female rats were interleaved. (*S*)-DHPG was purchased from Hello Bio Inc. (Princeton, USA). All statistical analyses were performed using GraphPad Prism 9.0.

In each experiment, submaximal field excitatory postsynaptic potentials (fEPSPs) were evoked by setting stimulus intensity to three times the threshold for eliciting a visually detectable response. Baseline stimulus intensities (15–30 µA) were similar for all age groups. LTD was induced after 30 min of stable fEPSP baseline recording by bath application of (*S*)-DHPG (50 µM, 10 min). fEPSPs were quantified by their initial slopes, and the level of LTD relative to baseline was measured in the last 10 min of the recording period (50–60 min after DHPG washout). Slices from juvenile female and male rats exhibited similar levels of DHPG-LTD of 21.5% ± 3.0% (*n* = 6/6) and 20.5% ± 2.1% (*n* = 8/8), respectively (unpaired *t*-test: *t*(12) = 0.3, *p* = 0.8; Figure 1(a)). Similarly, slices from young adult female and male rats did not differ in their levels of DHPG-LTD (25.3% ± 2.5% (*n* = 5/5) and 23.1% ± 1.9% (n = 6/6)), respectively; unpaired *t*-test: *t*(9) = 0.8, *p* = 0.5; Figure 1(b)). In contrast, slices from older adult female rats had less DHPG-LTD (27.2% ± 3.2%; *n* = 9/9) than their male counterparts (43.5% ± 3.9%; *n* = 9/7; unpaired *t*-test: *t*(16) = 3.3, *p* = 0.005; Figure 1(c)). Further analysis of the data considering both sex and age suggested that this difference can be attributed to an age-dependent increase in the level of DHPG-LTD in males that is absent in females (two-way analysis of variance (ANOVA), interaction effect of age and sex: *F*(2, 37) = 6.0, *p* = 0.005). Specifically, slices from older adult male rats exhibited significantly enhanced DHPG-LTD compared to slices from juvenile (*p* < 0.0001) and young adult (*p* = 0.0001) males (with no difference (*p* = 0.8) between male juveniles and young adults), whereas levels of DHPG-LTD did not differ between any of the female age groups (older adult versus juvenile: *p* = 0.4; older adult versus young adult: *p* = 0.9; juvenile versus young adult: *p* = 0.7; Tukey’s multiple comparisons test; Figure 1(d)).

**Figure 1.**
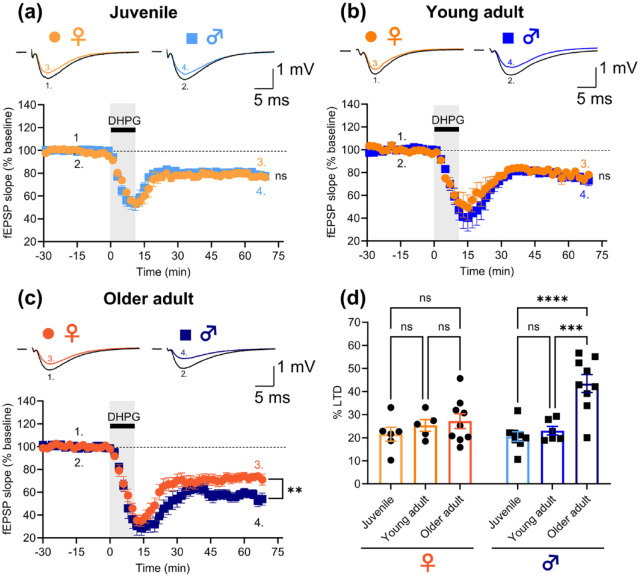
DHPG-LTD is enhanced in older adult male but not female rats. The magnitude of DHPG-LTD is not significantly different between (a) female (*n* = 6/6) and male (*n* = 8/8) juvenile nor (b) female (n = 5/5) and male (n = 6/6) young adult rats. (c) Older adult male rats (*n* = 9/7) exhibit increased DHPG-LTD in comparison to older adult female rats (*n* = 9/9). (d) Quantification of the levels of DHPG-LTD in slices from female and male rats. DHPG-LTD does not differ between any age group in female rats, whereas older adult male rats have increased DHPG-LTD in comparison to juvenile and young adult male rats. Representative example traces in (a)–(c) are averages of sweeps from the entire baseline period (1. and 2.) and the last 10 min of the recordings (3. and 4.) in female and male rats, respectively. Stimulus artefacts have been blanked for clarity. Pooled data are shown as mean ± SEM. Datapoints in the bar graph (d) represent the level of LTD measured in individual hippocampal slices. Unpaired *t*-tests were used to compare male vs female data in (a)–(c) and Tukey’s test was used for the pairwise comparisons in (d) (ns: not significant, **p < 0.01, ***p < 0.001, ****p < 0.0001; detailed statistical results are given in the main text).

Synaptic plasticity may, to some extent, be influenced by the basal properties of synaptic circuits (Tidball et al., 2017). Therefore, we also compared input/output (I/O) function and paired-pulse facilitation (PPF) in slices from male and female rats within each age group. Levels of pre-synaptic axon activation and basal synaptic transmission were assessed by varying the stimulus intensity (Figure 2(a)–(c)) and plotting the fibre volley (FV) amplitude as a function of stimulus intensity (Figure 2(d)–(f)), and the fEPSP slope as a function of FV amplitude (Figure 2(g)–(i)), respectively. PPF was assessed by delivering paired stimuli across a range of inter-pulse intervals (50, 100, 200 and 500 ms; Figure 2(j)–(o)). We found no differences in any of these parameters between male and female rats within each of the age groups, indicating that the specific enhancement of DHPG-LTD in older male rats is not due to a sex difference in basal synaptic function (unpaired *t*-tests for female vs male juvenile (*n* = 10/10 and 10/10), young adult (*n* = 6/6 and 6/6) and older adult rats (*n* = 10/5 and 10/5), respectively for (1) the stimulus-FV relationship: *t*(18) = 0.2, *p* = 0.8; *t*(10) = 0.3, *p* = 0.8; *t*(18) = 1.3, *p* = 0.2; (2) the FV-fEPSP relationship: *t*(18) = 1.0, *p* = 0.4; *t*(10) = 0.3, *p* = 0.8; *t*(18) = 0.08, *p* = 0.9; and (3) PPF: *t*(18) = 0.5, *p* = 0.6; *t*(10) = 0.6, *p* = 0.6; *t*(18) = 1.0, *p* = 0.3). It was noted, however, that there was a trend for increased basal synaptic transmission and decreased PPF with age in both sexes, which is consistent with previous findings (Pereda et al., 2019).

**Figure 2.**
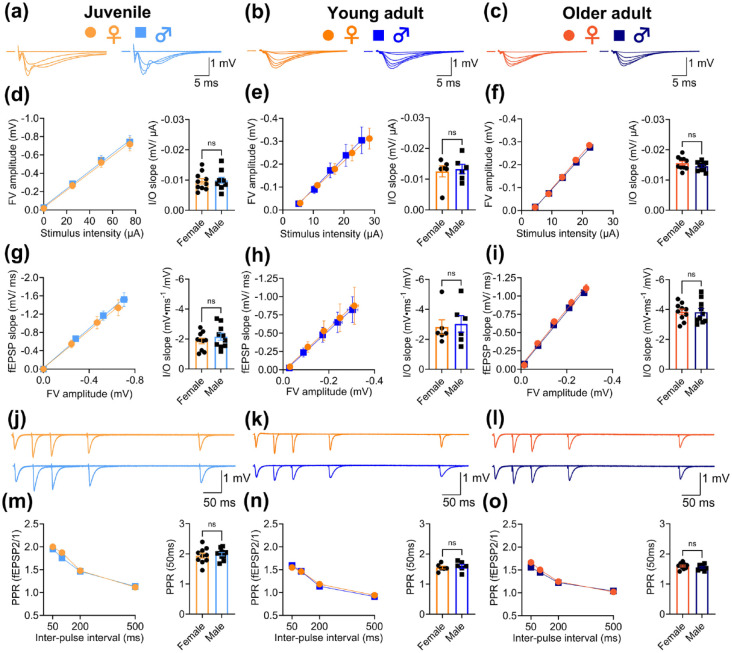
Basal synaptic properties do not differ between female and male rats in any age group. There were no differences in (d)–(f) the stimulus intensity to FV amplitude relationship, (g)–(i) the FV amplitude to fEPSP slope relationship, nor (m)–(o) PPF in female (*n* = 10/10) versus male (*n* = 10/10) juvenile (left panels), female (*n* = 6/6) versus male (*n* = 6/6) young adult (centre panels) and female (*n* = 10/5) versus male (*n* = 10/5) older adult (right panels) rats. I/O curves in juvenile rats were performed at fixed stimulation intensities (0, 25, 50 and 75 µA), whereas threshold multiples (1×–5×) were performed in young adult and older adult rats. I/O relationships were quantified by linear regression (slope values of the linear fits in individual slices are shown in the bar graphs). PPF in all age groups was assessed across a range of inter-pulse intervals (50, 100, 200 and 500 ms), and quantification of the paired-pulse ratio (PPR) in individual slices at the 50-ms interval is shown in the bar graphs. Representative traces (superimposed) for input/output curves in (a)–(c) and PPF experiments in (j)–(l) are averages of four consecutive sweeps. Stimulus artefacts have been blanked or truncated for clarity. Pooled data are shown as mean ± SEM and each datapoint in the bar graphs represents one hippocampal slice. Male versus female comparisons within each age group were made using unpaired *t*-tests (ns: not significant; detailed statistical results are given in the main text).

The present observation that there is a greater magnitude of DHPG-LTD in older male rats agrees with a previous report (Kumar and Foster, 2007). In contrast to males, we saw no increase in the level of DHPG-LTD with age in slices from female rats. The underlying mechanism for this sex difference is unknown. DHPG-LTD is a complex phenomenon, which has both pre- and post-synaptic loci of expression (Sanderson et al., 2022) and involves multiple signalling pathways, any one of which could be regulated in a sex-dependent manner. Of note, estradiol can influence signalling pathways via a direct interaction of oestrogen receptors with group I mGluRs (Meitzen et al., 2012). It is also interesting that 17β-estradiol and DHPG depress inhibitory post-synaptic currents in the hippocampus via mutually occlusive mechanisms (Tabatadze et al., 2015).

The magnitude of mGluR-LTD observed in hippocampal slices is likely to depend on the differential contribution of various induction and expression mechanisms (Tidball et al., 2017). In the previous study in males (Kumar and Foster, 2007), the enhanced DHPG-LTD in slices from older rats was associated with a shift in induction mechanisms, including an increased involvement of mGlu1 and N-methyl-d-aspartate (NMDA) receptors, and a reduced dependence on protein phosphatase activity. Whether such a shift in induction mechanisms with age is absent in females remains to be determined. In the same study, enhanced expression of DHPG-LTD was also attributed, at least in part, to heightened excitability in the CA3 region, since the difference between older and younger male rats was attenuated when the CA3 region of the slice was removed. Interestingly, in our experiments with intact slices (CA3 attached), we observed fluctuations in the level of LTD in some recordings, which we believe may be attributable to spontaneous hyperactivity of CA3 inputs triggered by DHPG treatment (Merlin and Wong, 1997; Young et al., 2013). This effect occurred predominantly (though not exclusively) in slices from older male rats. Thus, an increased propensity for mGluR-mediated hyperexcitability may be one mechanism underlying the specific enhancement of DHPG-LTD in older adult males.

Whatever the underlying mechanism, our results show that DHPG-LTD can be used to investigate sex-dependent differences in mGluR signalling, which may be relevant both to physiological brain function and pathological conditions (Lüscher and Huber, 2010). The findings of enhanced DHPG-LTD in the hippocampus of older males, but not females, are of particular importance to clinical treatments that are being developed for neurodegenerative conditions impacting cognitive function. Furthermore, while we detected differences in DHPG-LTD only in older adult rats, it is possible that sex differences in mGluR function could manifest earlier in development under certain pathological conditions, including neurodevelopmental disorders such as autism. Therefore, future studies of mGluR-LTD in animal models of neuropathology should consider both sex and age.
